# Framework for smartphone-based grape detection and vineyard management using UAV-trained AI

**DOI:** 10.1016/j.heliyon.2025.e42525

**Published:** 2025-02-06

**Authors:** Sergio Vélez, Mar Ariza-Sentís, Mario Triviño, Antonio Carlos Cob-Parro, Miquel Mila, João Valente

**Affiliations:** a JRU Drone Technology, Department of Architectural Constructions and I.C.T., University of Burgos, Burgos, 09001, Spain; b Information Technology Group, Wageningen University & Research, Wageningen, 6708 PB, the Netherlands; c Atos IT Solutions and Services Iberia, 28037, Madrid, Spain; d Centre for Automation and Robotics (CAR), Spanish National Research Council (CSIC), 28006, Madrid, Spain

**Keywords:** YOLO, Yield mapping, Precision agriculture, Vineyard management, Real-time detection, Digital agriculture

## Abstract

Viticulture benefits significantly from rapid grape bunch identification and counting, enhancing yield and quality. Recent technological and machine learning advancements, particularly in deep learning, have provided the tools necessary to create more efficient, automated processes that significantly reduce the time and effort required for these tasks. On one hand, drone, or Unmanned Aerial Vehicles (UAV) imagery combined with deep learning algorithms has revolutionised agriculture by automating plant health classification, disease identification, and fruit detection. However, these advancements often remain inaccessible to farmers due to their reliance on specialized hardware like ground robots or UAVs. On the other hand, most farmers have access to smartphones. This article proposes a novel approach combining UAVs and smartphone technologies. An AI-based framework is introduced, integrating a 5-stage AI pipeline combining object detection and pixel-level segmentation algorithms to automatically detect grape bunches in smartphone images of a commercial vineyard with vertical trellis training. By leveraging UAV-captured data for training, the proposed model not only accelerates the detection process but also enhances the accuracy and adaptability of grape bunch detection across different devices, surpassing the efficiency of traditional and purely UAV-based methods. To this end, using a dataset of UAV videos recorded during early growth stages in July (BBCH77-BBCH79), the X-Decoder segments vegetation in the front of the frames from their background and surroundings. X-Decoder is particularly advantageous because it can be seamlessly integrated into the AI pipeline without requiring changes to how data is captured, making it more versatile than other methods. Then, YOLO is trained using the videos and further applied to images taken by farmers with common smartphones (Xiaomi Poco X3 Pro and iPhone X). In addition, a web app was developed to connect the system with mobile technology easily. The proposed approach achieved a precision of 0.92 and recall of 0.735, with an F1 score of 0.82 and an Average Precision (AP) of 0.802 under different operation conditions, indicating high accuracy and reliability in detecting grape bunches. In addition, the AI-detected grape bunches were compared with the actual ground truth, achieving an R^2^ value as high as 0.84, showing the robustness of the system. This study highlights the potential of using smartphone imaging and web applications together, making an effort to integrate these models into a real platform for farmers, offering a practical, affordable, accessible, and scalable solution. While smartphone-based image collection for model training is labour-intensive and costly, incorporating UAV data accelerates the process, facilitating the creation of models that generalise across diverse data sources and platforms. This blend of UAV efficiency and smartphone precision significantly cuts vineyard monitoring time and effort.

## Highlights

• AI framework with a 5-stage pipeline integrating UAV-trained models for real-time grape bunch detection using smartphones
• X-Decoder refines segmentation, isolating grapevines from background, boosting YOLOv8’s accuracy in fruit detection.
• Scalable AI solution using UAV data to customize and speed up model training, ensuring adaptability and generalization
• Trained on UAV videos for fast, tailored data collection, enabling data generalization with smartphone images.
• Introduced a web-based AI tool for farmers to analyze vineyard images and estimate grape yield using smartphones.

## 1 Introduction

The global population's growth demands increased agricultural production for food security. Precision Agriculture (PA) is key to enhancing crop yield and quality using data and automation. PA's role in viticulture, which is significant for its economic and cultural value, is noteworthy. Precision viticulture applies PA to improve vineyard outcomes, focusing on maximising yield and minimising environmental harm. It uses local soil, climate, and vine health data for better management practices [1]. The deployment of new sensors for vineyard monitoring is expected to increase rapidly in the coming years, providing substantial information for better vineyard management [2].

This information can be efficiently processed by deep learning algorithms for applications such as the automatic classification of plant health and the identification of specific diseases [3], water stress [4] or yield management and the detection of fruits [[5], [6], [7]]. Among the array of deep learning methods, You Only Look Once, or YOLO [8], stands out significantly, and several authors have explored its application in fruit detection. Thus, in the broader agrifood sector, YOLO-based frameworks have been applied to a variety of fruits. For olive detection [9], showcased YOLO's effectiveness in improving the accuracy of olive fruit detection on trees [10]. utilised YOLOv4 for high-precision orange detection in orchards, while [11] classified mandarin oranges into quality categories using YOLO V2. The adaptability of YOLO was also demonstrated by Refs. [12,13] in mango, by Ref. [14] for oil palm fruit detection, and by Ref. [15] for pineapple bud detection, with enhancements aimed at mobile and Unmanned aerial vehicle (UAV) image processing. In tomato (Ge et al., 2022), applied the YOLO-Deepsort network for tomato identification and counting, showcasing high precision across various tomato maturity stages, thus demonstrating YOLO's broad applicability in the agricultural sector for yield estimation, quality classification, and enhancing postharvest processes. For pears [16], utilised YOLOv4 models for real-time pear counting in mobile applications, highlighting YOLO's versatility across different fruit types and detection scenarios, and [17] combined YOLO with Kalman filter for detecting pears and apples in video recordings, achieving high precision under variable conditions. Apple detection has been broadly explored, and several authors have employed various iterations of the YOLO framework to enhance accuracy and efficiency in different situations [18]. improved Yolov7 with self-attentive mechanisms, integrating multi-object tracking to accurately detect and count apples in images of apples within complex environments. Also [19], optimised YOLO-v5 for real-time recognition of apple stem/calyx to improve postharvest commercialisation processes. Similarly [20], explored YOLO among other methods for creating an automatic system for apple yield estimation in orchards, while [21] introduced an improved YOLOv7-tiny model for robust apple detection in challenging environments. Other authors have also further contributed to this field by enhancing apple detection through various YOLO architectures, ranging from YOLO v3 to YOLO v7, demonstrating significant advancements in precision, real-time processing, and yield estimation [18,[22], [23], [24], [25], [26]].

Despite the existence of these multiple YOLO models, their practical application, especially the latest YOLOv8 [27], has seen limited integration. Nevertheless, some studies have begun to unlock its potential in agricultural applications. For instance Ref. [28], implemented MHSA-YOLOv8 to enhance the grading and counting of tomato maturity, showcasing significant improvements in accuracy and efficiency over traditional manual methods, even under varying environmental conditions. Similarly [29], developed LS-YOLOv8s, incorporating the LW-Swin Transformer to boost feature extraction and classification capabilities for detecting and grading strawberry ripeness, aiding in automating strawberry picking and orchard management. Moreover [30], presented a method utilising YOLOv8 alongside a tracking-by-detection framework with NanoDet for counting and estimating the ripeness levels of cherry tomato bunches, showing potential for precision agriculture within greenhouse settings. Further extending YOLOv8's utility [31], introduced YOLOv8-GP, a model designed for the simultaneous detection of grape clusters and picking points, aiming to augment the efficiency of automated picking robots and facilitate their deployment on mobile robots.

Yet not only are algorithms essential, but the hardware utilised plays a vital role as well. On the one hand, smartphones have emerged as indispensable tools for farmers, seamlessly integrating into their daily routines. These devices not only facilitate immediate access to crucial data such as weather forecasts, market prices, and agricultural advisories but also enable the deployment of farming apps that can guide decision-making processes in real-time [[32], [33], [34], [35]]. On the other hand, UAVs are at the forefront of precision viticulture for the management of crop variability, and have shown potential in vineyards for applications such as yield estimation [36,37], phenotyping [38], vegetation monitoring [39,40], fruit quality assessment [41], nutrient analysis [42], disease detection and mapping [[43], [44], [45]] and water stress estimation [46,47]. Moreover, advancements in AI-based UAV swarms have further expanded their utility in agriculture [48]. Their sensors provide fast and accurate monitoring of the vineyard, essential for modern vineyard management [49,50], and can collect massive quantities of data in a very short time [51].

This transition to more advanced technologies has enabled more precise monitoring of crop health and the environment, leading to more informed decision-making in vineyard management. However, while these technologies promise enhanced productivity, their adoption is often hindered by the need for specialized hardware, making them less accessible to small-scale farmers. The growing adoption of smartphones in agriculture [52], combined with AI-driven models trained on UAV data, offers a promising solution, bridging the gap between high-tech innovations and practical, scalable applications in everyday agricultural practices.

Therefore, this study presents a five-stage AI Framework that integrates UAV-based training with smartphone-based grape detection. The pipeline uses X-Decoder for referring segmentation and YOLOv8-seg for instance segmentation to accurately detect grape bunches in smartphone images. A web application was developed to facilitate the easy integration of this AI framework with mobile technology, providing farmers with a cost-effective and user-friendly tool for real-time vineyard monitoring.

The remainder of this paper is organized as follows: The "Materials and Methods" section details the experimental setup, data acquisition, and the proposed AI framework. The "Results" section presents the performance metrics of the proposed model and its application in real-world scenarios. The "Discussion" section interprets the findings, addresses the limitations, and suggests future research directions. Finally, the "Conclusion" summarizes the contributions of this research and its potential impact on precision viticulture.

## 2 Materials and Methods

### 2.1 Experiment location

The research was conducted in July 2022 in two commercial vineyards in Tomiño, Spain, which grow Vitis vinifera cv. Loureiro grapes. These vineyards are owned by "Bodegas Terras Gauda S.A." in Pontevedra, Spain. The vineyards, named Vineyard B7 (coordinates X: 517204.2, Y: 4645059.6) and Vineyard B9 (coordinates X: 517016.7, Y: 4644818.4), are located in the UTM zone 29N (ETRS89/EPSG: 25829), are part of the "Rías Baixas DO" area, which means they follow strict rules and practices. These vineyards have a northeast-southwest orientation and 196.17C rootstock, chosen for their suitability in humid conditions and resistance to active limestone. Between the vine rows, a variety of natural vegetation grows spontaneously. The vines were in the phenological stage BBCH77-BBCH79 [53], and the training system used here is vertical shoot positioning, supported by a vertical trellis. The vineyards were initially planned with plant and row spacing of 2.5 × 3 m. In terms of vineyard management, no artificial alterations like leaf removal are done. This approach keeps the natural look of the grape bunches, which is evident in the images and videos showing the typical leaf occlusion found in the vineyards. It is important to note that some plants were affected by Esca disease to assess the framework's robustness against varied health states.

### 2.2 Datasets

#### 2.2.1 UAV data acquisition and preprocessing

**Fig. 1:**
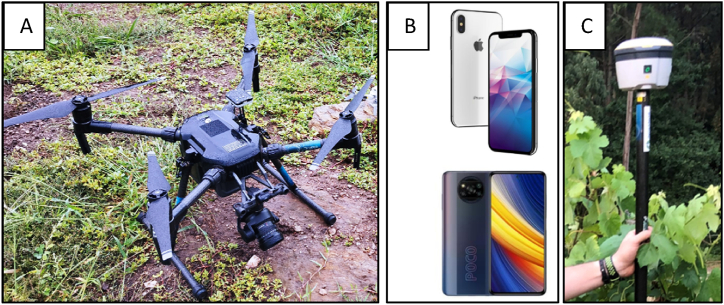
Equipment Used in the Study. A. DJI M210 Drone. B. Smartphones - iPhone X (top) and Xiaomi Poco X3 Pro (bottom). C. Trimble R^2^ Integrated GNSS System.

This study used a DJI M210v2 drone (Fig. 1A), fitted with a DJI Zenmuse X5S sensor. This drone is a multi-rotor platform known for its robustness and versatility. The high-resolution camera has a 20.8-megapixel sensor and a pixel size of 3.4 μm, capable of recording videos at 4096 × 2160 pixels. This equipment was chosen for its ability to capture detailed images from a stable platform. The UAV flights took place over the vineyards on June 28th, 2021, flying 3 m above the ground, capturing the vineyard rows closely for detailed observation. The weather was optimal, with clear skies and wind speeds below 0.5 m/s, ensuring stable flight conditions.

**Fig. 2:**
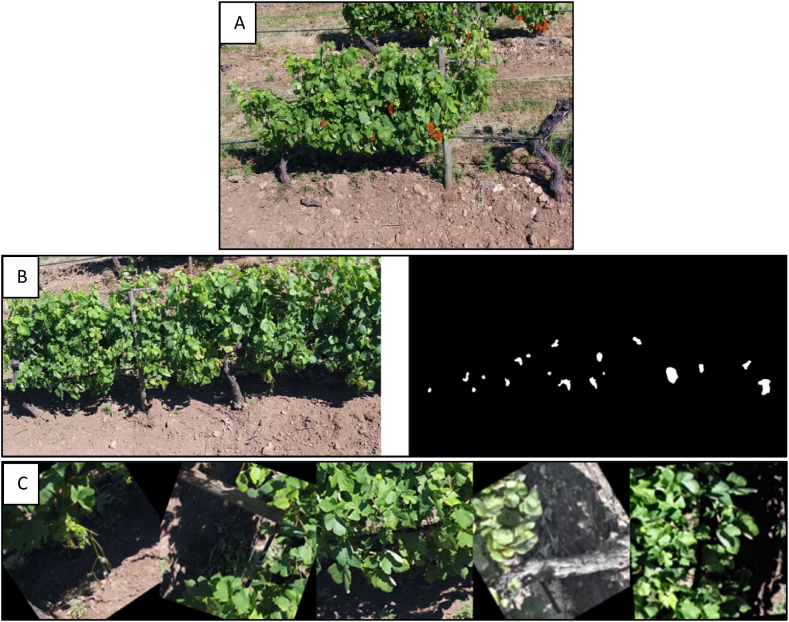
A) Annotations produced with CVAT software of the grape bunches (Adapted from Ref. [38]). B) Example of a pair of images after adjusting grayscale levels. C) Example of the final images used in the training after the augmentation process.

In total, 40 RGB videos were recorded across four flights, each covering one side of a vineyard row. These videos, amounting to 7.49 gigabytes, provided a comprehensive view of the grape clusters at different developmental stages, from BBCH75 (pea-size) to BBCH79 (bunch closure). The camera was tilted at 60°, capturing extensive views of the grape clusters. The CVAT software (CVAT.ai Corporation, 2022) was used with the MOTS technique (Fig. 2A) for detailed analysis. Expert agronomists annotated the grape clusters in 29 video sequences, resulting in 679 frames with per-pixel accuracy. The annotations focused on the grape clusters, excluding the peduncle, and included clusters in varying light and location conditions. The videos were then split approximately 70/30 for training and testing purposes. The complete dataset, along with the MOTS labels for the grape clusters, is publicly available [54]. This availability follows the FAIR principles (Wilkinson et al., 2016), guaranteeing that datasets and methods are easily accessible and useable, supporting reproducibility, collaboration and further research in precision agriculture.

In order to transform the data into a correct YOLO structure that could be used to train a new model, a series of preprocessing steps were applied.

1. Grayscale Adjustment: the pair of images was adjusted to an appropriate grayscale to enhance the visibility of grapes (Fig. 2B).
2. Image resizing: the images were resized, optimising the dimensions for further processing.
3. Patching Process: a patching process was performed, consisting of splitting the image into small ones, overlapping patches, and re-merging the patches to avoid losing the correct segmentation information.
4. Translation to Bounding Boxes: the annotated segmentation information was translated into bounding box formats compatible with YOLOv8-seg.
5. Data Augmentation: Techniques such as horizontal and vertical flips, scale and rotation transformations, and colour and light adjustements were applied using the Albumentations library (Buslaev et al., 2020) to increase dataset size and diversity. These were applied randomly over the images (Fig. 2C) to obtain better results in the training process.
6. Split: Lastly, a split of the pictures between the training and the validation was performed.

#### 2.2.2 Smartphone photos acquisition

In 2022, images from each plant were captured by two different users using two smartphones alternatively: the Xiaomi Poco X3 Pro (Xiaomi Corporation, Beijing, China) and the iPhone X (Apple Inc., Cupertino, USA). These models (Fig. 1B) represent typical consumer-grade mobile phones and were specifically chosen for their relevance to farmers' everyday devices. The Xiaomi Poco X3 Pro features a 48 MP primary camera, a 6.67-inch display, and a Snapdragon 860 chipset, while the iPhone X has a 12 MP primary camera and is powered by the Apple A11 Bionic chip. These mobile phones were chosen due to their operating systems; one is based on Android (Xiaomi Poco X3 Pro) and the other on iOS (iPhone X), the two main operating systems in mobile phones, which cover a vast majority of the smartphone market. They symbolise the accessibility and utilisation of modern smartphone technology by farmers increasingly adopting digital tools for agricultural practices [52]. The dataset is available at [55].

**Fig. 3:**
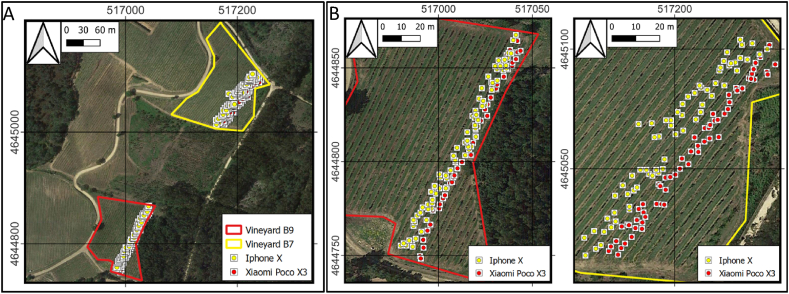
A) Location of the vineyards. B) Photo locations within each vineyard, captured by the smartphones without high-precision GNSS correction. CRS ETRS89/zone 29N.

A total of 253 images (Fig. 3) of grape bunches were captured from two different vineyards under varying lighting conditions (midday and afternoon) to test the framework in diverse outdoor lighting scenarios, critical for accurate grape bunch detection and counting. Due to the landscape, the very nature of the vineyards, and other factors, the plants die from pests, diseases, or physiological problems of the plant itself [56], so a certain number of plants must be replanted each year. In fact, the physiology of a young plant is very different from that of an adult plant, so regulatory bodies do not consider these plants to be in full production [57]. Therefore, these plants were not used for the experiment.

#### 2.2.3 GNSS trunk position acquisition

While convenient, the consumer-grade smartphones used in the study lacked precise geolocation capabilities. Therefore, a Trimble R^2^ Integrated GNSS system (Fig. 1C) with a TSC3 Controller (Trimble Inc., California, USA) was employed to pinpoint the location of each plant accurately. The dataset can be found at [58]. This system provides centimetre-level accuracy, far exceeding the typical GPS capabilities of a standard smartphone and adds a layer of valuable spatial data. As a result, highly accurate yield maps could be generated. Since the locations of the trunks remain constant over time, this precise location data needs to be collected only once or perhaps every few years.

### 2.3 Proposed framework

**Fig. 4:**
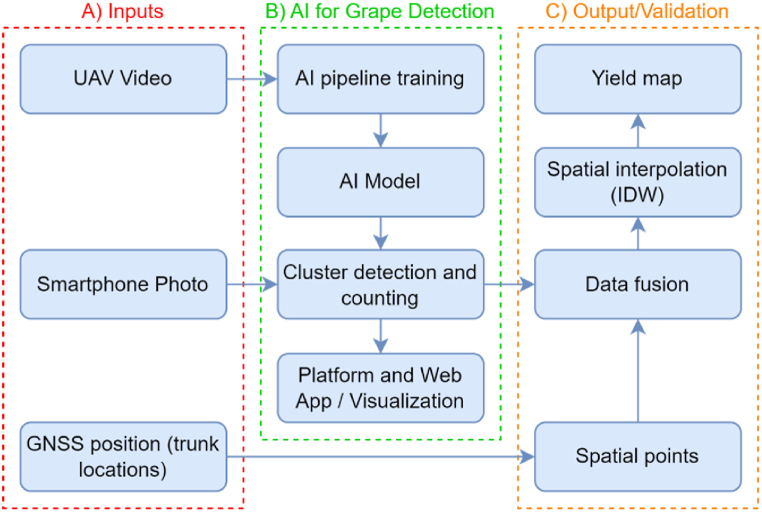
Integrated Framework for Grape Yield Estimation. A) Data inputs, including UAV video, smartphone photos, and GNSS position data. B) AI-driven process for grape detection, including model training, cluster counting, and web-based visualisation. C) Generation of a yield map through Inverse Distance Weighting (IDW) spatial interpolation and data fusion.

Fig. 4 shows the proposed framework, a streamlined process combining UAV, smartphone data and AI. UAVs quickly capture field videos for AI model training. This trained AI then analyses smartphone images for AI-driven grape detection and counting. Simultaneously, GNSS locates vine trunks, matching these locations with AI data. Finally, this integrated data is converted into a yield map, providing vital insights into cluster quantity and distribution for effective vineyard management. Below, each stage is described in detail.

1. X-Decoder Application: This model was employed for initial segmentation, differentiating grapevines from the background. The X-Decoder supports various vision-language tasks and was pre-trained on a mixed dataset with limited segmentation data.
2. Grape Zone Extraction: The segmented images were further processed to extract specific zones potentially containing grape clusters, ensuring that the subsequent stages focus on relevant areas.
3. Patching and Image Preparation: The grape zones were divided into patches to optimise the detection process by improving the aspect ratio of the clusters.
4. YOLOv8-Seg Implementation: The YOLOv8x-seg model, known for its balance between speed and accuracy, was used for instance segmentation. We trained this model on the UAV dataset, using a batch size of 8, an image size of 640 pixels, and the SGD optimiser. The training ran for 150 epochs on an Nvidia A5500 GPU. Data augmentation was applied to improve the model's generalisation.
5. Reassembly and Segmentation Mask Generation: After detection, patches were reassembled into a full segmentation mask, providing a comprehensive view of the detected grape clusters.

#### 2.3.1 AI for grape detection

**Fig. 5:**
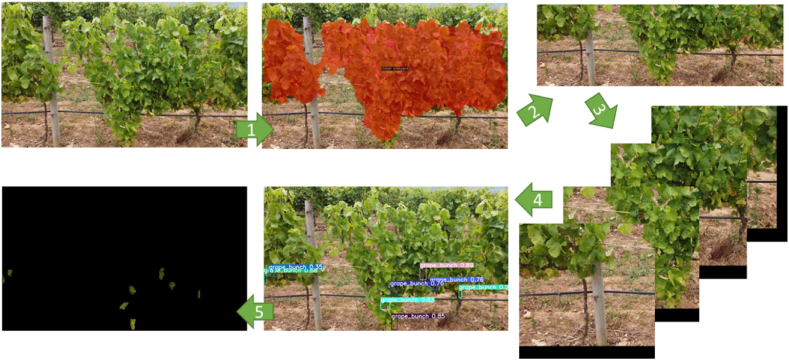
AI 5-step AI Pipeline for Grape Detection: 1) X-Decoder separates vines from the background, 2) extracts grape zones, 3) patches are processed, 4) YOLOv8-seg detects clusters, and 5) patches are reassembled into a segmentation mask.

Aiming to ensure precise detection and counting of grape clusters (AI pipeline training, AI model and Cluster detection and counting, in Fig. 4), a 5-step process for AI grape detection was developed (Fig. 5): The method initiates with raw vineyard data as input, followed by the application of the 1) X-Decoder model for referring segmentation differentiates between foreground vines and other image elements, such as the ground, sky, or even background vines. Subsequently, the process involves 2) the extraction of specific grape zones from this segmented image, yielding cropped images focused solely on areas potentially containing grape clusters. These specific cropped zones then undergo thorough 3) a patching process in order to improve the aspect ratio of the grapes clusters to prepare the images for 4) using the YOLOv8-seg model, where each patched image is individually processed to detect and accentuate grape clusters. The final stage of this pipeline 5) reassembles the patches and reconstructs a single segmentation mask from the resulting polygons of YOLOv8-seg.

X-Decoder is a novel model [59] that integrates pixel-level and image-level vision-language understanding, seamlessly supporting various types of image segmentation and vision-language (VL) tasks. The X-Decoder was pre-trained on a mixed dataset containing limited segmentation data and millions of image-text pairs and follows the generic design of encoder-decoder architecture but is distinguished by three critical design features: (1) it incorporates both latent and text queries, producing semantic and pixel-level outputs; (2) a single text encoder is employed to process a wide range of text corpus, including class concepts, referring phrases, and image captions; (3) the image and text encoders are decoupled to facilitate cross-image tasks such as image-text retrieval, as well as within-image tasks like segmentation and captioning. As a result, the X-Decoder can be utilised to unify a diverse array of vision and vision-language tasks. These include generic segmentation, encompassing instance, semantic, and panoptic segmentation with support for open-vocabulary and zero-shot scenarios; referring segmentation, which identifies specific image segments based on arbitrary textual queries provided by the text encoder; image-text retrieval, achieved through the extraction of decoupled image and text representations and the use of dot-product calculations to determine similarities; and image captioning, wherein textual tokens are decoded using the same autoregressive decoder. In this work, Referring Segmentation was employed, which requires both latent and text queries as inputs and achieves state-of-the-art results across eight datasets, with competitive or better performance on segmentation and VL tasks compared to other generalist and specialist models.

The YOLO (You Only Look Once) framework is known for its balance of speed and accuracy in real-time object detection. It has been applied in various fields, such as autonomous vehicles, robotics, video surveillance, augmented reality, agriculture, and facial recognition systems [60]. Unlike other object detection methods that use a sliding window approach or region proposal networks to detect objects in an image, YOLO works by dividing an image into a grid of cells and predicting bounding boxes and class probabilities for each cell, treating object detection as a single regression problem and allowing it to process the entire image in one forward pass. This capability makes YOLO significantly faster than other object detection algorithms while maintaining high accuracy. Since its inception, the YOLO family has evolved through multiple iterations, improving performance and addressing limitations with each new version.

Furthermore, the advanced YOLOv8 model [27] was employed specifically for this research, recognised for its state-of-the-art performance in object detection tasks [61]. It is the latest iteration in the YOLO series, distinguished for its effective balance between inference speed and accuracy, which is crucial for real-time applications. This model analyses images in a single pass, making it exceptionally fast and efficient. Its design enables the accurate detection of objects even in challenging conditions, such as varying sizes, speeds, and backgrounds. Therefore, the architecture of YOLOv8 is particularly adept at handling high-variability tasks, making it an ideal choice for detecting grape clusters in diverse vineyard environments. In addition, from the suite of models in the YOLOv8 family, the YOLOv8x-seg model was specifically chosen for training with the video dataset. Two key factors drove this decision. Firstly, the model's segmentation capabilities were selected over detection alone in anticipation of future applications to assess the health status of grape bunches, where accurate differentiation of the bunch from the vine's foliage is crucial. Secondly, the absence of hard real-time constraints permitted the selection of the largest model in the YOLOv8-seg lineup, enabling more comprehensive knowledge and superior metrics from the training data if overfitting is avoided.

Unlike traditional segmentation models, YOLOv8-seg does not rely on segmentation masks during its training phase. Instead, this model adopts another approach by using labelling formats like those in object detection tasks but with a significant modification: It utilises standard bounding box annotations but augments them with contour details through inner bounding polygons, resulting in a label per seg-instance like: <class_id> <bbox_coords> <bpolygon_cords>. This way, YOLOv8-seg merges the benefits of object detection, such as speed and efficiency, with those of segmentation, notably the precision in outlining object contours. This fusion makes YOLOv8-seg exceptionally well-suited for applications demanding accurate delineation of object boundaries without incurring the computational costs associated with pixel-level segmentation.

The precision (Equation (1)) and recall (Equation (2)) of the model were evaluated:

Eq. (1) $$Precision=\frac{TP}{TP+FP}$$

Eq. (2) $$Recall=\frac{TP}{TP+FN}$$

Eq. (3) $$IoU=\frac{Area\;of\;Intersection}{Area\;of\;Union}$$

with TP representing true positives, FP false positives, and FN false negatives. In instance segmentation, predictions are classified as true positives (TP) when the segmentation mask or polygon aligns with the ground truth above a predetermined IoU threshold (Equation (3)). The terms mAP50 or mAP@0.5 refer to this IoU criterion. This standard asserts that segmentation instances are considered accurate if they overlap with the ground truth masks by at least 50 %, as determined by the IoU metric. Finally, the F1 score was calculated (Equation (4)), a composite metric that balances precision and recall:

Eq. (4) $$F1=2\times \frac{Precision\times Recall}{Precision+Recall}$$

### 2.4 Platform and web App

This system has been deployed and tested on a platform where, through open-source technology, a Kubernetes cluster was deployed to host all the AI tools and the web application, as detailed in Ref. [62]. In this environment, the project's pipelines are executed and put into production, including the web application. The FlexiGroBots web application can be accessed at [63] and is designed for ease of use, only requiring drag-and-drop images acquired with smartphones. This straightforward method shows the effectiveness and practicality of the web app in real-world situations where mobile assistance is utilised, and its web-based naturity could facilitate its real implementation, as evidenced in other studies [64].

**Fig. 6:**
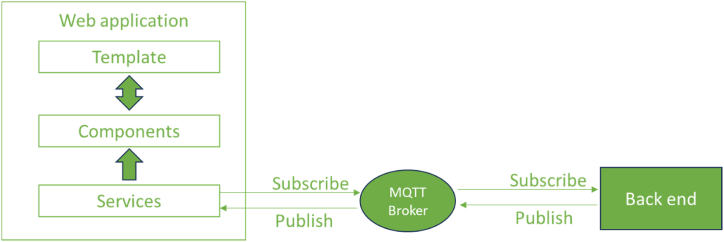
Basic schema of the FlexiGroBots web application infrastructure.

**Fig. 7:**
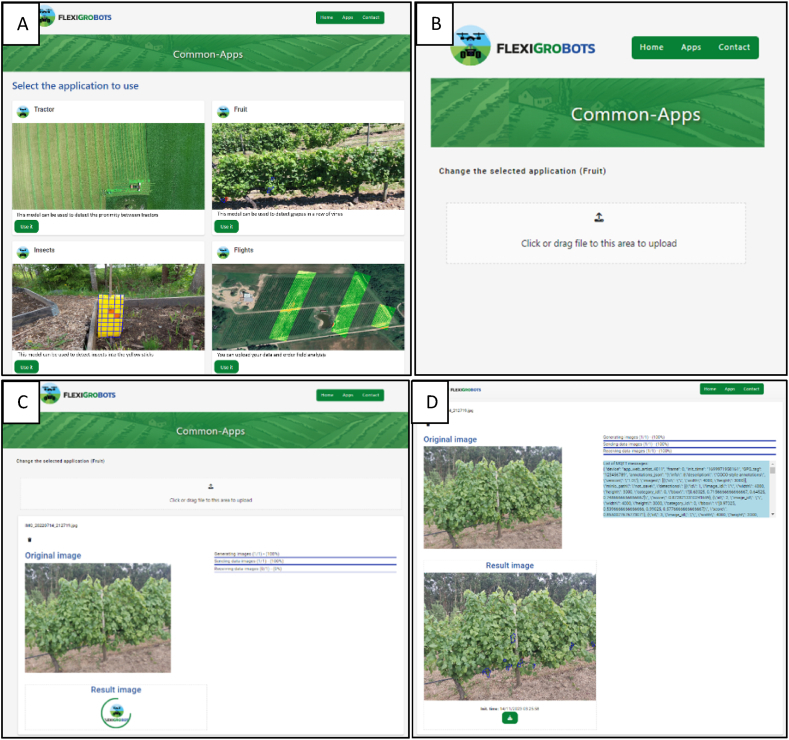
FlexiGroBots web application. A) Landing page. B) Fruit counting app. C) Uploading and processing the image. D) Results.

In order to enhance the front-end experience and, therefore, the interaction between the final user and the overall system, the FlexiGroBots web application was developed using a single-page application (SPA) approach. This method moves away from the traditional page reload mechanism (Fig. 6), making the web application more responsive and similar to a native app. The foundation of this application is built on the Angular framework [65], and it is organised into three main layers: core, abstraction, and presentation. While the specifics of each layer are not the focus here, it is essential to mention that logic has been integrated into these layers to properly manage the consumption of topics from the Message Queuing Telemetry Transport (MQTT) server. Since the back-end communication relies on topics published to an MQTT broker, the front-end must effectively differentiate topic consumption. This differentiation must account for concurrent back-end requests, which could come from various users or multiple requests by the same user through different web browsers, ensuring efficient and correct data handling at the front-end. Fig. 7A displays the web application's landing page, the FlexiGroBots platform's main gateway, and the suite of AI agricultural tools.

The web application features a simple layout with five modules: "Tractor", "Fruit", "Insects", "Weeds", and "Flights", each with specific functions like tractor proximity detection, grape detection, insect identification on traps, weed detection and field analysis data upload. Each module includes a description and a "Use it" button.

The "Fruit" counting application (Fig. 7B) within the FlexGroBots platform is designed for user-friendly interaction. The interface is clean and minimalistic, featuring a large, prominent upload area where users can click or drag files to input their data. Above this upload area is a link allowing users to change the selected application, indicating that the "Fruit" application is active. In Fig. 7C, the functionality of the application "Fruit" counting is further elaborated, showing the image upload and processing stages. In this example, the user uploaded an image, as indicated by the file name "IMG_20220714_212719.jpg", above the progress bars. These bars provide real-time status updates on the process: image generation, data sending, and data reception, with the image generation and sending processes marked as complete when reaching 100 %. Below the progress indicators, the interface displays the "Original image" section, where the uploaded vineyard photo is visible and ready for analysis by the platform algorithms. There is also a placeholder for the "Result image", where the analysed image with detected fruit will be displayed once the processing is complete.

In Fig. 7D, the result of the "Fruit" counting application is depicted, following the processing steps of the server. The image, initially uploaded by the user, has been analysed, and the results are now displayed. The progress indicators show that all stages—image generation, data sending, and data reception—are complete at 100 %. The interface showcases the "Original image" at the top and the "Result image" below. In the "Result image", grape clusters have been identified and marked with blue overlays, indicating successful detection by the algorithms. Accompanying the result is a timestamp labelled "Init. time" that provides the exact time of the analysis completion, which adds a layer of traceability and precision to the process.

Additionally, the interface includes a log of MQTT messages, tracking the technical communication between the device and the server. These messages contain metadata such as frame identification, timestamps, GPS tags, and annotations in JSON format that describe the detected objects with their respective coordinates, dimensions, and scores. This metadata can be helpful for users requiring detailed analytical information, as it enables a deeper understanding of the image processing results and offers insights into the precision and reliability of the detections made by the AI model.

### 2.5 Cross-validation

Finally, to validate the methodology, a visual count ("Visual method") of grape clusters in the images was compared with those identified by the algorithm. This data was then correlated with the GNSS positions of the plants recorded by the GNSS receiver, leading to the creation of yield maps by Inverse Distance Weighting (IDW) spatial interpolation for visual comparison. Furthermore, for statistical analysis, plant-specific values were assessed to evaluate the accuracy of the grape cluster detection algorithm and the proposed method. The analysis was conducted in groups: firstly, examining all plants collectively, and secondly, focusing on plants without Esca disease to assess the impact of the disease. The results were validated using a *t*-test to check for significant differences.

R^2^ (coefficient of determination) and RMSE (Root Mean Square Error) were used to verify the statistical accuracy of the data. R^2^ measures the proportion of variance in the dependent variable predictable from the independent variable, while RMSE provides the standard deviation of the prediction errors or residuals, offering insight into the accuracy of the prediction model. Additionally, to enrich the dataset, a comparison was made with ground truth (GT) data collected directly from the field to determine the degree of leaf occlusion in the vineyard. Exhaustive data from a total of 42 plants, encompassing 449 grape clusters, were included in this analysis.

## 3 Results

**Fig. 8:**
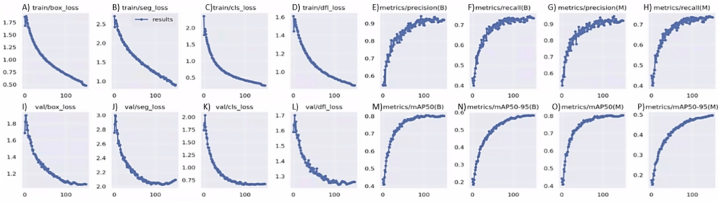
Performance metrics evolution for YOLOv8x-seg model training and validation for 150 epochs. The graphs labelled with (B) represent bounding box metrics and detection tasks, while those marked with (M) correspond to segmentation mask metrics.

The grape bunch detection pipeline was delineated into five well-defined phases, leveraging two AI models: X-Decoder for referring segmentation and YOLOv8x-seg for instance segmentation. The reflected results will focus on the performance metrics derived from the training of the YOLOv8x-seg model and evaluate the holistic effectiveness of the entire detection pipeline. This result is due to the referring segmentation task, which does not require additional training or fine-tuning, hence assuming the results reported in the original X-Decoder paper are representative [59]. Fig. 8 illustrates the training process of the YOLOv8x-seg model on the dataset outlined in section 2.2, undergoing training for a total of 150 epochs with a batch size of 8, an image size of 640 pixels, and utilising the SGD (Stochastic Gradient Descent) optimiser. The model was trained on a single Nvidia A5500 GPU with 16 GB of vRAM for over 25 h.

During its training phase, the model showed the expected progression, achieving stability in its performance metrics. Towards the end of the training, the decline in all the loss functions became minimal, never reversing direction but approaching a horizontal asymptote (Fig. 8A–D). Therefore, it was decided not to train the model for additional epochs to avoid the adverse effects of overfitting. The model's precision was notably high at 0.92 (Fig. 8E and G), highlighting its accuracy in identifying grape bunches. Recall, a metric that evaluates the model's ability to detect all relevant instances within the dataset, was recorded at 0.735 (Fig. 8F and H), representing the percentage of actual grape bunches that the model correctly identified among all possible detections. The F1 score was 0.82. This score indicates an effective equilibrium between precision and detection capability, demonstrating the model's robust performance in recognising grape bunches within the dataset under review. This score is especially useful in scenarios where the balance between precision and false negatives is crucial.

**Fig. 9:**
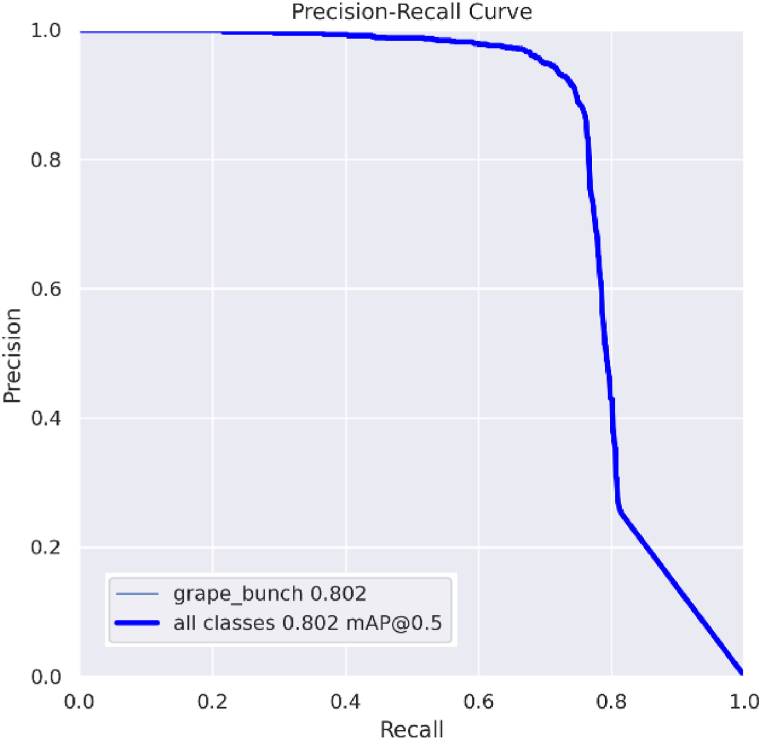
Precision-Recall Curve for segmentation task (M) on the "grape bunch" class at a mAP-0.5 IoU threshold.

The area under the Precision-Recall curve (Fig. 9) results in an Average Precision (AP) score of 0.802, which, in this context, equates to the mean Average Precision (mAP) due to the evaluation being limited to a single class "grape-bunch". This AP value and the previous metrics highlight the model's proficiency in accurately detecting grape bunches.

**Fig. 10:**
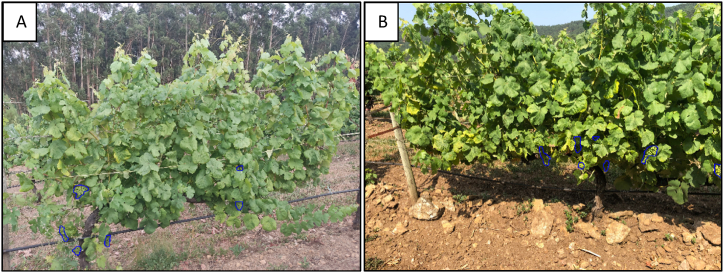
Model detection outcomes in smartphone images, highlighted by a blue perimeter. A) Non-favourable Lighting Conditions (Evening). B) Favourable Lighting Conditions (Midday).

The AI system was able to pinpoint clusters of grapes on the vines with precision. Fig. 10 displays the detection results of the model on smartphone images, with the detected areas outlined in blue under poor lighting conditions captured in the evening (Fig. 10A) and good lighting conditions gathered around midday (Fig. 10B). The images present a landscape many vine growers would recognise, with brown, rocky soil typical in vineyards and rows of vines supported by the vertical trellis. This setup includes a wooden post that secures the trellis wire and additional wires that support the plants. Black tubing is visible at the base, a part of the drip irrigation system that slowly waters the vines.

**Fig. 11:**
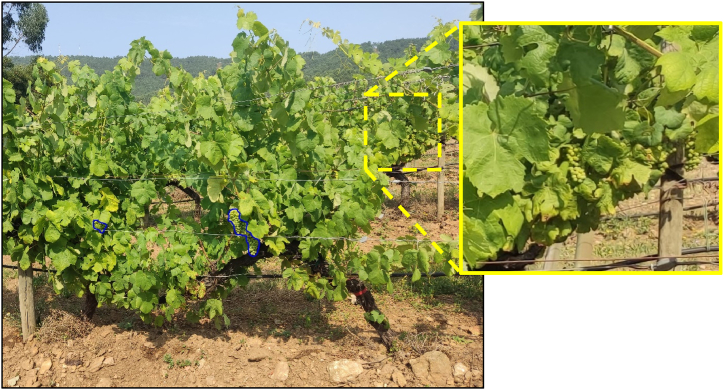
Thanks to the previous segmentation with X-Decoder, the model only detects grape bunches in the first row but not in the background rows.

Fig. 11 exhibits the outcome of the AI model's detection process, following segmentation with the X-Decoder technique, which allows the model's selective detection in the spatial plane. The image demonstrates the model's ability to discern grape clusters in the foreground, highlighted with blue outlines, but it does not detect similar clusters in the vine rows in the background. An inset, framed by a yellow border, offers an enlarged view of the vines in the background, providing an example of the ability of the model to recognise only the grape clusters at the front amidst the complex backdrop of leaves and branches.

### 3.1 Cross-validation with ground truth data

**Fig. 12:**
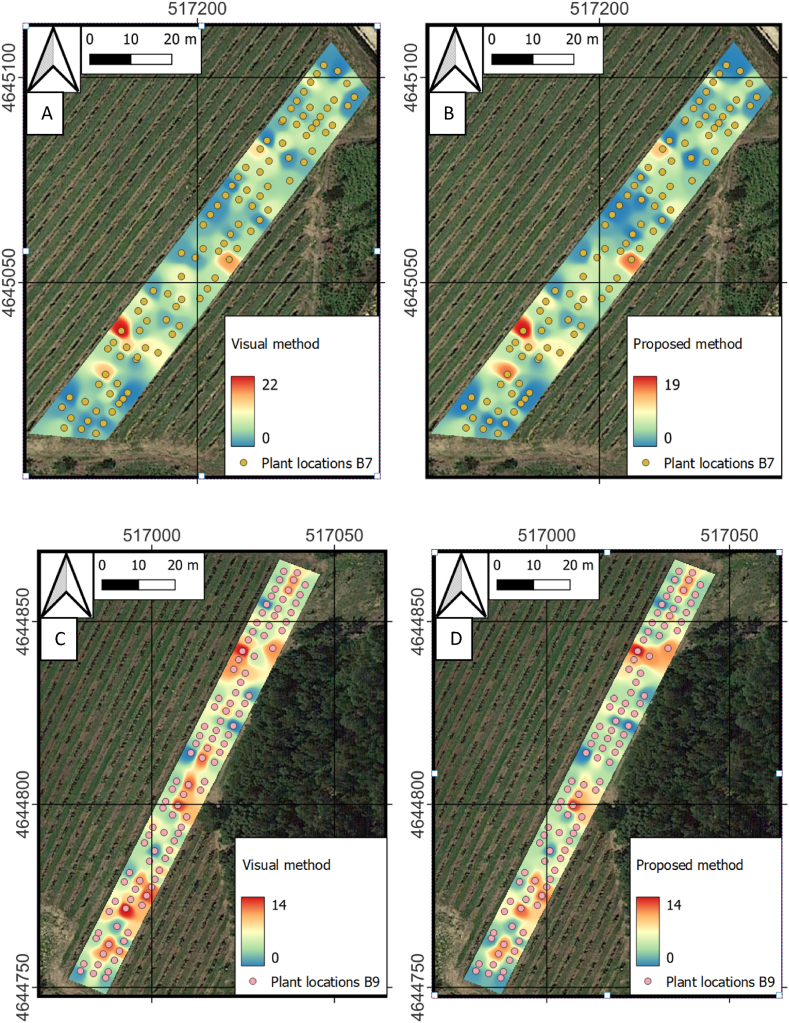
Yield maps generated from the number of bunches counted on the images. Maps A and B are located in vineyard B7, whereas C and D are in vineyard B9. Maps A and C were produced employing the values derived from the "Visual method", and B and D were created utilising the methodology introduced in this research.

Further processing, Fig. 12 shows the yield maps derived from GNSS and AI pipeline data fusion. It is composed of four distinct maps. Maps A and B are located in vineyard B7, whereas C and D are in vineyard B9. Furthermore, Maps A and C were produced employing the values derived from the visual method, and B and D were created utilising the methodology introduced in this research. The results derived from visual inspection and the proposed method are represented as heat maps superimposed on satellite images of the vineyards. It can be observed that the maps are extremely similar, following the same visual patterns. Nevertheless, there are slight discrepancies between the counts of the visual method and the algorithm, with the former systematically higher in cluster number in the middle and upper zones of the map. While both methods identify similar trends in the spatial distribution of clusters, the proposed method seems to slightly underestimate yield compared to the visual method.

**Fig. 13:**
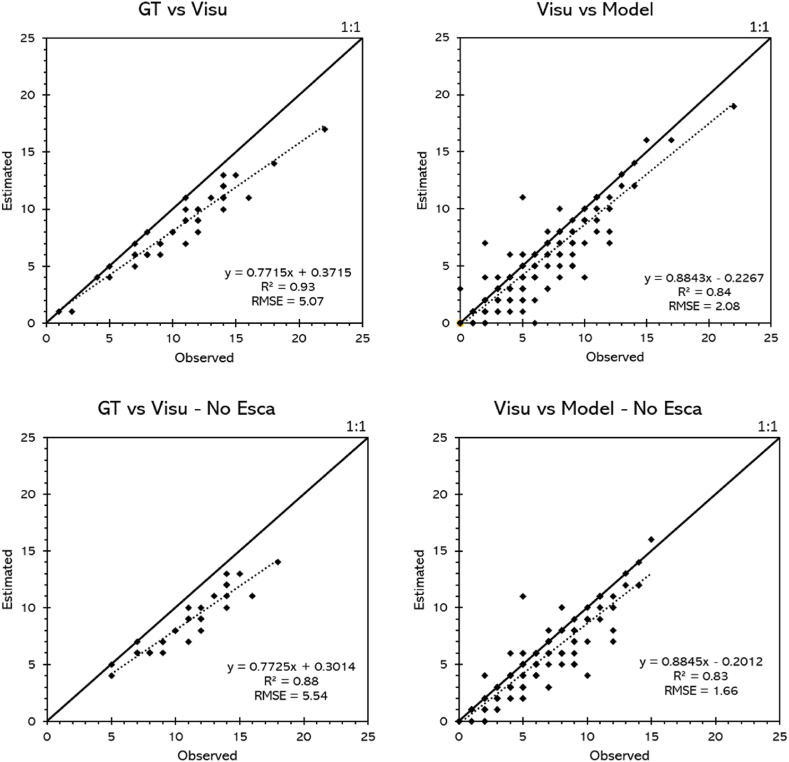
Comparison between the actual count of grape clusters in a vineyard (GT), manual counting from photographs (Visu), and detection by a computational algorithm (Model), with and without the inclusion of clusters affected by Esca disease.

In order to validate the results, Fig. 13 presents a comparative analysis of performance evaluation methods in vineyards, using grape cluster counting as the primary metric. It displays four scatter plots contrasting cluster count estimates and observations in a vineyard. The top charts show data for all plants, while the lower charts exclude data from plants affected by Esca disease. On the left side, the comparison of Ground Truth (GT) against visual estimation (Visu) is shown, while on the right side, visual estimation (Visu) against the model prediction (Model) is represented. The difference between the total number of grape clusters (GT) and the number of clusters visually detected in the images by the expert was assessed, finding an average occlusion rate of about 20 %. In the top left chart (GT vs Visu), a strong correlation is observed with an R^2^ of 0.93 but with a trend line slope of 0.7715, indicating that the visual estimation tends to be systematically lower than the real one. This makes sense since clusters completely covered by vegetation cannot be visually discerned in the images. In the bottom left (GT vs Visu - No Esca), the correlation remains strong with an R^2^ of 0.88 and a similar slope of 0.7725, suggesting that excluding plants with Esca does not significantly change the pattern of visual underestimation. Comparing Visu against the model (top-right chart), the computational model shows a slope of 0.8843 with an R^2^ of 0.84, indicating good correlation, though with a slight tendency of the model to underestimate the count compared to the visual estimation, mainly due to difficulty in identifying partially occluded clusters. Excluding Esca (bottom-right chart), the slope adjusts slightly to 0.8845 with an R^2^ of 0.83, maintaining a solid correlation and confirming that Esca does not significantly affect the model's prediction. Additionally, a *t*-test between plants with and without Esca reflected that this disease does not substantially impact cluster detection (p-value <0.05). In the same way, no significant differences (p-value <0.05) were found in the detection accuracy of grape clusters between images collected in the afternoon and midday and in the photos collected using different phones.

The RMSE in GT vs Visu is 5.07, while in Visu vs Model, the RMSE decreases to 2.08, indicating that the accuracy of the model is greater between the values estimated by the proposed model and the visual ones than between the visual ones and the GT. That is, the impact of clusters covered by vegetation is greater than the error of the proposed model compared to the count made on the images by a professional. This improvement is maintained when excluding plants with Esca, where the RMSE further decreases to 1.66 in Visu vs Model - No Esca, implying that the model is slightly more precise when not considering plants affected by this disease.

## 4 Discussion

Estimating the number of grape clusters per plant is crucial because this variable is one of the components of the vineyard yield and directly impacts grapevine productivity [66]. For this purpose, consumer-grade mobile phones can be effective. The web app introduced in this article demonstrates practical and cost-effective solutions for grape bunch detection and counting, highlighting the opportunities for farmers to utilise the tools already at their disposal, specifically smartphones, to adopt technology for more efficient and effective agricultural management [33]. By integrating drones, smartphones, and geostatistical analysis, this approach seeks to close the gap between high-tech solutions and their application in agriculture to make precision farming tools more accessible to growers. To the best of our knowledge, this is the first time such a framework has been presented.

The research found a higher count of grape bunches in the field (GT) compared to those visible in photos (Visu), attributable to approximately 20 % of bunches occluded by the foliage, hiding them from sight. Despite this minor obstruction, the discrepancy is considered negligible [67]. In addition, given that many bunches are located on the lower portion of the vines, and the aim was to include as many vines as possible, images were captured from only one side of each vine. Capturing both sides might have revealed more bunches, but the primary focus of this research is on developing the innovative framework rather than quantifying foliage coverage, so this limitation does not significantly impact the study objectives.

The maps demonstrate the capability of the proposed framework to produce data that closely match the ground truth (Fig. 12), a fact confirmed by the statistical results, with strong correlations between Ground Truth (GT) and visual estimations (Visu), and between visual estimations and model predictions (Model), with R^2^ values up to 0.93 and 0.84 respectively. Yet, the AI pipeline underestimates the number of bunches slightly compared to the visual method. This observation is supported by statistical findings, indicating a consistent underestimation in visual counts relative to GT due to occlusions and a minor model underestimation against Visu, with RMSE metrics revealing superior model precision (2.08) over GT versus Visu (5.07). Both methodologies, however, reveal similar patterns in the spatial arrangement of grape clusters, aligning with the findings of other researchers that highlight YOLO as an effective means for evaluating the spatial variability of grape yield in vineyards [6,68]. Remarkably, the presence of Esca disease seemingly has no substantial impact on the trend of underestimation or model forecasts, as evidenced by comparable slopes and R^2^ figures in analyses omitting plants afflicted by Esca. These findings are essential for vineyard management, particularly since climatic changes impact yield. Inaccuracies in yield predictions could notably impact essential decisions regarding harvesting, resource allocation, and the logistics of sales [69]. Precise estimation of vineyard yields is critical for enhancing both efficiency and profitability in viticulture since this aspect is a crucial component of effective vineyard management [70]. The method proposed in this study shows an advance in the automation of this process.

While numerous algorithms have showcased efficacy in grape detection, our project has distinctively and specifically integrated YOLO into a five-stage AI pipeline (Fig. 5), primarily due to its proven effectiveness, as highlighted by various researchers. Some authors have effectively utilised models such as MobileNet-V1, SSD Inception-V2 and Swin Transformer-Tiny, showcasing their capabilities in identifying grape bunches at different growth stages with remarkable precision [71,72]. However, despite these developments, the selection of YOLO for our project is based on its reliable and real-time processing capabilities, as evidenced by the research conducted by Ref. [68], who evaluated YOLOv3, YOLOv3-tiny, YOLOv4, YOLOv4-tiny, YOLOv5x, YOLOv5s for real-time detection and counting of white grape bunches, and [73], who developed GA-YOLO, based on YOLOv4, to assess grape yield spatial variability in a vineyard. Moreover, our results are consistent with those of other authors who have used YOLO to detect occluded grapes [74]. Other authors have also used YOLO (v3 and v5) to detect grape clusters with high accuracy and speed successfully [7,75]. However, the inclusion in our AI pipeline of the latest version of the algorithm, YOLOv8, is a breakthrough that improves accuracy and efficiency in specific viticulture applications. The research by Ref. [76] on using YOLOv8 for fruit ripeness evaluation, although not explicitly addressing grapes, demonstrates the improved analytical power of the new version, showcasing its relevance in the broader spectrum of agricultural studies.

Therefore, a notable strength of the method introduced here lies in the integration of YOLOv8 within the AI pipeline (Fig. 5), which, unlike previous approaches that relied on bounding boxes for object detection [68,75,[77], [78], [79]] instead captures the precise contours of grape clusters, potentially facilitating more accurate yield estimations by allowing for the direct measurement of cluster areas [38]. achieved a similar level of detail in grape outline delineation, albeit through the application of YOLACT and an improved Mask R-CNN model, with a precise segmentation and enumeration of grape berries, effectively addressing the visual complexities posed by overlapping clusters and occlusion within grape imagery. Our results demonstrate that this approach significantly enhances the detection accuracy of grape bunches, achieving a high precision of 0.92 and an F1 score of 0.82, indicating a well-balanced performance between precision and recall. Specifically, the model achieved a mean Average Precision (mAP) of 0.802 for the "grape-bunch" class, as evidenced by the area under the Precision-Recall curve. Additionally, in challenging lighting conditions, such as those in the evening, the model maintained robust detection capabilities, as shown in Fig. 11. This research aligns with the findings of other studies that highlight the effectiveness of YOLOv8, marking a significant step forward in accuracy and efficiency compared to traditional methods [[28], [29], [30]]. Specifically, within viticulture, the outcomes are consistent with those of [31], who developed a YOLOv8-GP model that achieves high accuracy (AP: 89.7 %) for simultaneous detection of grape clusters and picking points to improve picking robots' efficiency.

In addition to YOLO, another significant strength of the method is the implementation of X-Decoder, which enhances the segmentation process by accurately distinguishing grapevines from complex backgrounds. Other researchers have addressed the challenge of background interference by using various approaches: capturing images at night with an artificial lighting system to reduce lighting variability and improve segmentation accuracy [80], employing a controlled background by placing a blue screen behind the plants during image collection [81], and utilising an RGB-D camera that captures both RGB images and depth information, applying depth threshold filtering to isolate the objects of interest [82]. However, these methods are not applicable in our context because our approach prioritizes interoperability and ease of use for the farmer. Moreover, as can be seen in the UAV dataset [54], flying UAVs between rows in commercial vineyards is challenging, often capturing several rows at once, which necessitates focusing on the first row of vegetation to improve training. Similarly, the smartphone dataset [55] reveals that, despite photos typically being taken at an appropriate height and nearly horizontal to the ground, images frequently capture background rows due to insufficient vegetation in the front row or gaps in the planting. Hence, X-Decoder plays a pivotal role in refining the initial segmentation, enabling the pipeline to concentrate on relevant areas of interest, such as the first row of vegetation, while effectively filtering out non-essential elements like additional rows or background noise. This approach ensures that subsequent stages of the pipeline, including YOLOv8's contour detection, work with cleaner, more relevant data, leading to more reliable yield estimations. The effectiveness of high-quality segmentation in improving object detection performance is evident in techniques similar to those found in X-Decoder, such as Deeplab [83] and Mask R-CNN [84]. The combination of X-Decoder for precise segmentation and YOLOv8 for detailed contour detection marks a substantial advancement in AI-driven solutions for precision viticulture, providing a more refined and effective tool for vineyard management.

Nevertheless, our framework diverges from these methodologies by not merely utilising YOLOv8 for detection but innovatively combining it in a five-stage AI pipeline with UAV video data to detect grape clusters in smartphone imagery. Other authors have also proposed a multi-stage pipeline to deal with unstructured background, occlusion and dense berries, for instance, proposing a two-stage pipeline with YOLOv4 for grape yield estimation, TSGYE, to detect grape clusters and efficient counting of berries [77]. However, the present methodology advances by exploiting UAV videos for input, significantly enhancing the ability of the model to adapt to the unique conditions of each vineyard quickly. Collecting images with smartphones for model training is both slow and expensive. However, adding UAV data speeds up this process and helps with the progress of machine learning by making it easier to create generalised models that work well with different kinds of data and on different platforms. This mix of UAV speed and smartphone detail can significantly lower the time and work needed to monitor vineyards. In addition, this flexible strategy enables swift retraining with fresh data from new vineyards, leading to a tailor-made solution adapted to the specific needs of each farmer, picturing a future where AI-driven solutions are not only accessible but also tailored to meet the complex demands of vineyard management. Furthermore, our framework.

An added benefit of the presented framework is its workflow, which outlines a method for producing initial yield maps utilising mobile phone images, devices that most farmers and viticulturists can easily access, making it easier to generate maps crucial for precise viticulture practices [40,45,[85], [86], [87], [88]]. The ultimate goal is to provide vineyard managers with reliable, affordable, automated tools to facilitate informed decision-making and optimise grape production processes.

### 4.1 Limitations and challenges

**Fig. 14:**
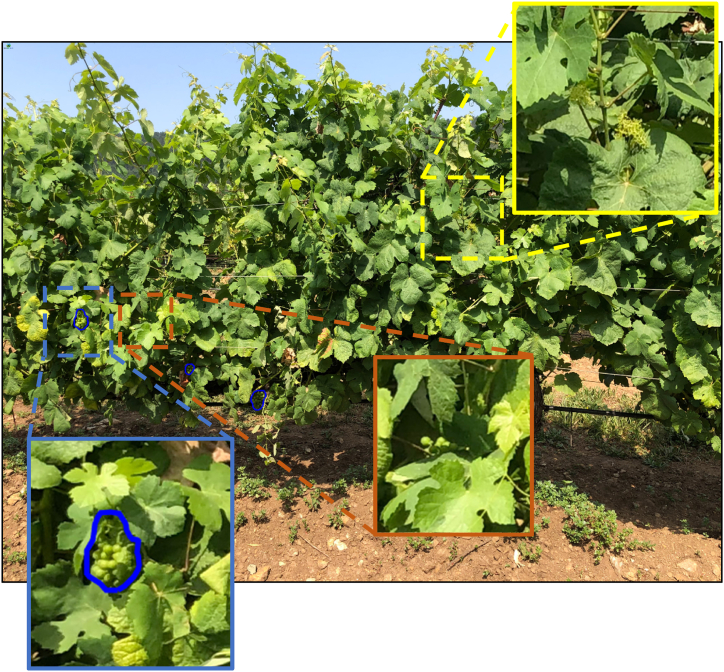
Limitations and challenges. Detection platform capabilities showcasing accurate differentiation between barren and fruitful bunches (yellow outline), alongside its ability to identify partially hidden grape clusters (blue), with occasional inaccuracies (brown) leading to underestimations.

In the endeavour of cluster detection and counting, the method has exposed specific limitations and challenges. Fig. 14 shows some details of the detection platform. A noteworthy advantage is the proficiency of the model in distinguishing barren bunches from fruitful ones, as highlighted in the yellow box. Conversely, the UAV-trained model exhibits the capability to identify even partially obscured grape clusters (marked in blue), though its accuracy fluctuates (indicated in brown), culminating in a tendency to underestimate (Fig. 13). The problem of leaf occlusion is well-documented in vineyards, making grape bunch counting difficult [89]. While some studies employ leaf removal to reduce occlusion and improve detection accuracy [37,77,90], this research maintains the natural state of the vineyards to better reflect the real-world conditions that farmers encounter daily in typical commercial vineyards, where such interventions are not always feasible. Similar challenges have been addressed by Ref. [79], who introduced an enhanced YOLO v4 model (YOLO v4+) tailored to tackle occlusion challenges in unstructured environments, introducing advancements like a parameter-free attention mechanism and a multi-scale feature fusion module, significantly elevating detection accuracy and the adaptability of the model in grape recognition.

**Fig. 15:**
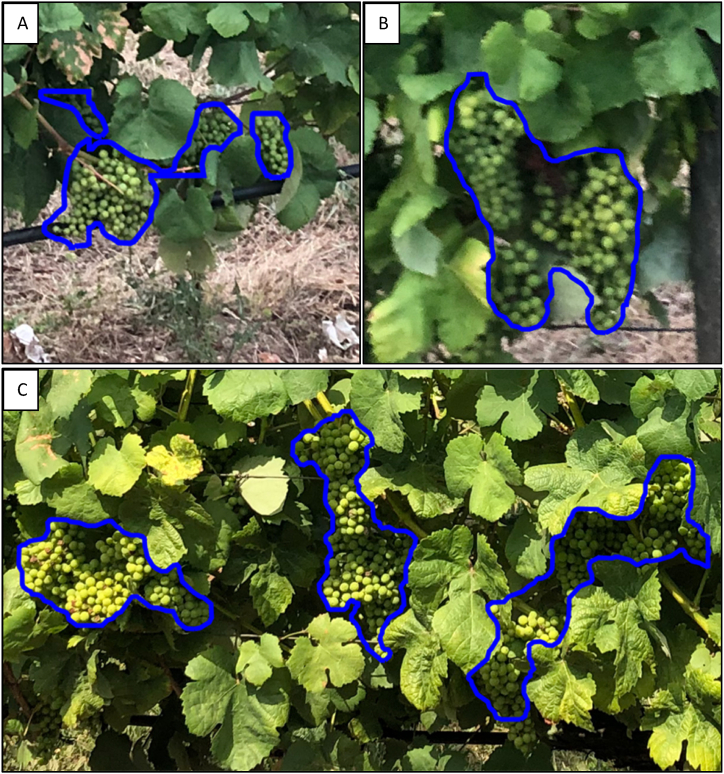
Model underestimation due to the consolidation of nearby grape clusters identified as one. A) and B) Images taken in the evening, and C) Image taken at midday.

**Fig. 16:**
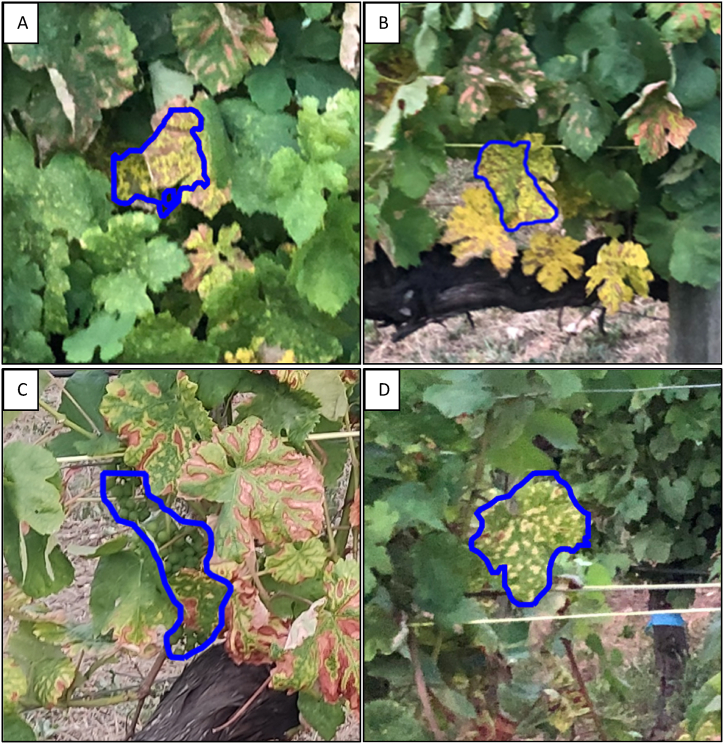
Challenges in detection are highlighted by false positives due to Esca disease-like patterns, resulting in overestimated cluster surfaces.

A further issue identified is the predisposition of the model to consolidate proximate grape clusters, perceiving them as a single entity. This phenomenon leads to a further underestimation of grape bunch counts. Fig. 15 presents instances of this problem, with the top image captured in the evening and the bottom one at midday. Conversely, instances of overestimation arise from misidentifying vegetation with colour patterns akin to grape clusters as actual fruit bunches (Fig. 16), though these errors are relatively infrequent, as indicated by the results.

**Fig. 17:**
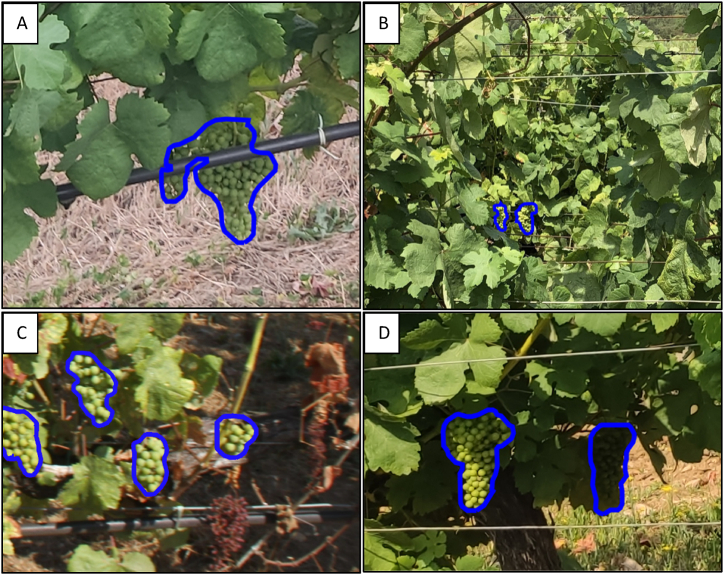
Examples of the successful detection of the model in challenging conditions: (a) a cluster beneath an irrigation pipe, (b) small bunches of green grapes densely covered with leaves, (c) correctly detected grape clusters and correctly undetected dry bunches, and (d) accuracy variations in sunny versus shaded bunches. The blue outlines indicate the detected perimeters.

Furthermore, it is particularly relevant to note that the model detects grape bunches under challenging conditions. Hence, Fig. 17A depicts a problematic scenario where a cluster of green grapes is beneath an irrigation pipe, a potentially problematic setting for detection, yet the model proficiently identifies and outlines the cluster with a blue contour amidst a background of mixed weeds and straw. Fig. 17B shows an image where vine foliage occludes two diminutive clusters of green grapes, which are challenging to discern due to vegetative density and hue. Moving to Fig. 17c, the photo shows a vineyard with several clusters of healthy green grapes in the fore, contrasted by visible soil and interspersed with dried bunches; the model aptly differentiates, recognising only the harvest-worthy green grapes and omitting the non-viable ones. Finally, Fig. 17d captures a sizeable cluster of green grapes alongside a shadowed cluster to the right, set against a confluence of soil and spontaneous vegetation. The model adeptly detects both clusters, effectively independent of their illumination levels.

Regarding leaf occlusion, it would be helpful to explore other vineyards with different levels of leaf occlusion to see how it affects accuracy. In this regard, specific YOLO models that could be useful to include have been developed for grape detection in these environments (J. Chen et al., 2023; Tian et al., 2019).

Future work can include upgrading the grape cluster application to not only detect and count but also to enable the model to identify grape clusters affected by diseases such as botrytis cinerea. In this sense [6], proposed a YOLO-based solution for detecting and classifying grape bunches as healthy or damaged, creating and using two datasets to better identify biophysical lesions in grape berries. In apples [91], propose a method using NIR cameras and a pruning-based YOLO V4 network for online apple defects detection, enhancing the efficiency of apple sorting in packing lines, and [92] introduced ALAD-YOLO, a lightweight model for detecting apple leaf diseases based on YOLO v5, achieving 90.2 % accuracy while reducing computational complexity. Thus, by employing the same logic as the framework outlined in this document, it is possible to generate prescription maps for disease treatment utilising merely smartphone images, similar to the methodologies suggested by other researchers [45,93]. Furthermore, considering the focus of this paper on early assessments, subsequent endeavours might include the identification of clusters at previous phenological phases, such as BBCH 71, marked by the onset of fruit swelling and the shedding of flower remnants [53], or even before, at peak bloom during BBCH 65, where half of the flower caps have dropped. This rationale aligns with existing research demonstrating the applicability of YOLO for recognising flowering stages, albeit in contexts other than vineyards [94,95].

## 5 Conclusions

This paper proposes an AI framework to improve grape detection within the context of precision viticulture. The framework includes using UAV videos to train the model, a 5-stage AI pipeline that is trained with the UAV videos, and deploying a web application to upload smartphone images so the farmers can detect and count the grape bunches in real time. Finally, with the plants' position and spatial interpolation, a yield map is generated to provide further information to the farmer.

X-Decoder, used in the framework, played a crucial role in accurately segmenting vineyard rows, effectively distinguishing the first veinayrd row from complex backgrounds and other rows. Combined with theYOLOv8x-seg model, this approach achieved a precision of 0.92 and recall of 0.735, with an F1 score of 0.82 and an Average Precision (AP) of 0.802, indicating high accuracy and reliability in detecting grape bunches within the dataset, underscoring the precision and utility of the proposed AI pipeline. Moreover, the smartphone image validation, with an R^2^ value reaching up to 0.84, revealed a high precision of the AI-detected grape bunches. The model was predisposed to underestimating the grape cluster count, primarily when faced with clusters positioned nearby or obscured by foliage. This tendency to merge neighbouring clusters into a singular count reflects a challenge inherent to viticulture. Given the prevalence of such conditions in vineyards, this area presents an opportunity for further refinement. Nevertheless, the ability of the model to discern grape clusters under challenging conditions such as partial occlusion or variable lighting illustrates its practical application in real-world settings. Furthermore, the inclusion of the UAV allowed for tailored training that considered this vineyard's unique characteristics, improving the system's accuracy in detecting grape clusters in various health and disease conditions. This adaptability is essential for technology to be truly transformative in the agricultural sector, showing the potential to impact the daily operations of farmers positively.

This research validates the framework as a potentially powerful tool in precision agriculture, leveraging UAV data to accelerate model training for a system primarily designed for smartphone use. By integrating YOLOv8 and X-Decoder, the framework significantly enhances vineyard monitoring, particularly in row-planted vineyards, offering a scalable, real-time solution for grape bunch detection, counting, and yield mapping. The adaptation of X-Decoder to these environments improves segmentation, making advanced technology practical and accessible for farmers using smartphones. This approach could potentially be adapted for other row-planted woody crops, extending its benefits beyond viticulture.

## CRediT authorship contribution statement

**Sergio Vélez:** Writing – original draft, Visualization, Validation, Software, Resources, Methodology, Investigation, Formal analysis, Data curation, Conceptualization. **Mar Ariza-Sentís:** Writing – review & editing, Validation, Methodology, Investigation, Conceptualization. **Mario Triviño:** Writing – original draft, Validation, Methodology, Investigation, Conceptualization. **Antonio Carlos Cob-Parro:** Writing – original draft, Validation, Methodology, Investigation, Conceptualization. **Miquel Mila:** Writing – original draft, Validation, Methodology, Investigation, Conceptualization. **João Valente:** Writing – review & editing, Validation, Supervision, Project administration, Methodology, Investigation, Funding acquisition, Conceptualization.

## Data availability

Data is available at https://doi.org/10.1016/j.dib.2024.110497 [55].

## Declaration of competing interest

The authors declare that they have no known competing financial interests or personal relationships that could have appeared to influence the work reported in this paper.
